# Exogenous Postharvest Application of Calcium Chloride and Salicylic Acid to Maintain the Quality of Broccoli Florets

**DOI:** 10.3390/plants11111513

**Published:** 2022-06-05

**Authors:** Hossam S. El-Beltagi, Marwa Rashad Ali, Khaled M. A. Ramadan, Raheel Anwar, Tarek A. Shalaby, Adel A. Rezk, Sherif Mohamed El-Ganainy, Samy F. Mahmoud, Mohamed Alkafafy, Mohamed M. El-Mogy

**Affiliations:** 1 Al Bilad Bank Scholarly Chair for Food Security in Saudi Arabia, The Deanship of Scientific Research, The Vice Presidency for Graduate Studies and Scientific Research, King Faisal University, Al-Ahsa 31982, Saudi Arabia; kramadan@kfu.edu.sa (K.M.A.R.); tshalaby@kfu.edu.sa (T.A.S.); arazk@kfu.edu.sa (A.A.R.); salganainy@kfu.edu.sa (S.M.E.-G.); 2 Agricultural Biotechnology Department, College of Agriculture and Food Sciences, King Faisal University, Al-Ahsa 31982, Saudi Arabia; 3 Biochemistry Department, Faculty of Agriculture, Cairo University, Gamma St, Giza 12613, Egypt; 4 Department of Food Science, Faculty of Agriculture, Cairo University, Giza 12613, Egypt; marwa3mrf@agr.cu.edu.eg; 5 Central Laboratories, Department of Chemistry, King Faisal University, Al-Ahsa 31982, Saudi Arabia; 6 Department of Biochemistry, Faculty of Agriculture, Ain Shams University, Cairo 11566, Egypt; 7 Postharvest Research and Training Centre, Institute of Horticultural Sciences, University of Agriculture, Faisalabad 38040, Pakistan; raheelanwar@uaf.edu.pk; 8 Department of Arid Land Agriculture, College of Agricultural and Food Science, King Faisal University, P.O. Box 400, Al-Ahsa 31982, Saudi Arabia; 9 Horticulture Department, Faculty of Agriculture, Kafrelsheikh University, Kafr El-Sheikh 33516, Egypt; 10 Plant Pathology Research Institute, Agricultural Research Centre, Giza 12619, Egypt; 11 Vegetable Diseases Research Department, Plant Pathology Research Institute, Agricultural Research Centre, Giza 12619, Egypt; 12 Department of Biotechnology, College of Science, Taif University, P.O. Box 11099, Taif 21944, Saudi Arabia; s.farouk@tu.edu.sa (S.F.M.); m.kafafy@tu.edu.sa (M.A.); 13 Vegetable Crops Department, Faculty of Agriculture, Cairo University, Giza 12613, Egypt

**Keywords:** *Brassica oleracea* var. *italica*, glucosinolates, shelf-life, storability, antioxidant activity, fresh-cut

## Abstract

The importance of broccoli (*Brassica oleracea* var. *italica*) consumption has increased in recent years due to its significant amount of anticarcinogenic and antioxidant compounds, as well as its many vitamins. However, broccoli florets are a highly perishable product which rapidly senesce and turn yellow after harvest, resulting in losses in nutritional and bioactive compounds. Thus, in this study, we evaluated the effect of postharvest exogenous of salicylic acid (SA) and calcium chloride (CaCl_2_) and their combination on the quality of broccoli florets stored at 5 °C for 28 days to minimize the rapid senescence of broccoli florets. Samples treated with 2 mM SA alone or in combination with 2% CaCl_2_ showed lower weight loss and lower losses of chlorophyll content, vitamin C, phenolic compounds, carotenoids, flavonoids, and glucosinolates compared with the control samples. Additionally, antioxidant activity was maintained by either SA or SA + CaCl_2_ treatments while peroxidase activity was decreased. For higher quality and lower losses in antioxidant compounds of broccoli florets during refrigerated storage at 5 °C, SA + CaCl_2_ treatment could be helpful for up to 21 days.

## 1. Introduction

Broccoli (*Brassica oleracea* var. *italica*) belongs to the *Brassicaceae* family, and it contains considerable levels of vitamins and antioxidants such as chlorophyll pigments, phenolic compounds, vitamin C, and glucosinolates [1]. In addition, previous studies indicated that the daily consumption of fresh broccoli could prevent cell damage that leads to cancer due to its high amount of sulforaphane [2]. However, broccoli is a highly perishable product due to its high water content and high respiration rate [3]. After harvest, broccoli florets senescence, and the green color degrades quickly, losing its commercial value [4] and nutritional content [5]. Thus, several previous approaches, such as the use of passive modified atmosphere treatment [6], exogenous sodium nitroprusside treatment [7], UV-C treatment [8], pulses of low-intensity light [9], and postharvest folic acid treatment [10], have been used to extend the shelf life and improve the quality of harvested broccoli heads. The previous techniques may not be suitable due to their high cost and/or difficulty in commercial applications. Thus, there is a need to find an effective and cheaply applicable technique for preventing senescence and maintaining nutritional compounds in harvested broccoli. The postharvest application of salicylic acid (SA) and calcium chloride (CaCl_2_) could be an alternative technique for maintaining the quality of broccoli heads during refrigerated storage. Moreover, SA and CaCl_2_ are classified as safe substances by the US Food and Drug Administration (FDA). Thus, both substances could be suitable for enhancing the quality and antioxidant compounds in broccoli.

Salicylic acid (SA) is a plant hormone that belongs to phenolic compounds in plants. SA is responsible for mitigating the abiotic stresses in plants, including salinity, drought stress, and chilling injury [11]. SA can also be applied safely to fruit postharvest. It has been reported that exogenous postharvest SA application delayed the repining and senescence of fruits and reduced weight loss and decay while increasing firmness [12]. In addition, Dokhanieh et al. [13] found an increase in the total phenolic compounds, flavonoids, anthocyanins, and ascorbic acid content of comelian cherry by dipping fruits in 2 mM SA after harvest. Additionally, the maintenance of previous compounds in litchi was obtained by dipping fruits in 1 mM SA after harvest [14]. Moreover, papaya and pear fruits immersed in 2 mM SA after harvest showed higher ascorbic acid content, antioxidants, phenolic compounds, and carotenoids than a control treatment during cold storage [15,16,17].

Calcium is an important element that is involved in several physiological operations in plants. Additionally, it helps to maintain the quality of horticultural products by maintaining the integrity of plant cells’ walls [18]. Thus, it plays a vigorous role in the quality, maturity, repining, and senescence of the horticultural products. One of the most famous soluble salt forms of calcium is CaCl_2_. Previous research has shown that calcium plays a role in the quality and the shelf-life of several vegetable crops, including cucumber [19] and strawberry [20]. Moreover, the application of calcium chloride after harvest delays the progress of ripening, reduces decay, and increases the calcium level in treated fruits, resulting in higher nutritional value in treated fruits [21,22]. Reducing respiration, ripening development, and senescence were the effects observed in some fruits and vegetables from CaCl_2_ application after harvest [23,24]. Moreover, Aghdam et al. [25] found that antioxidant capacity, anthocyanin, ascorbic acid, and phenolic compounds were conserved in cornelian cherry fruits by the postharvest application of CaCl_2_. Dipping fresh-cut sweet leaf bush in CaCl_2_ delayed yellowing and the degradation of chlorophyll content and maintained the total phenols and flavonoids [26].

However, little is known about the effects of postharvest SA and CaCl_2_ treatments on the quality of broccoli florets. To the best of our knowledge, this is the first report on the effect of SA, CaCl_2_, and their combinations on the quality of broccoli florets. Thus, the current work aims to evaluate the storage ability and quality of fresh-cut broccoli florets treated with SA, CaCl_2_, and their combinations. Weight loss, chlorophyll content, vitamin C, phenolic compound, carotenoids, flavonoids, glucosinolates, sulforaphane, and peroxidase activity were all measured in broccoli florets that had been sorted at 5 °C for 28 days.

## 2. Materials and Methods

### 2.1. Plant Material, Treatments, and Storage Condition

**Figure 1:**
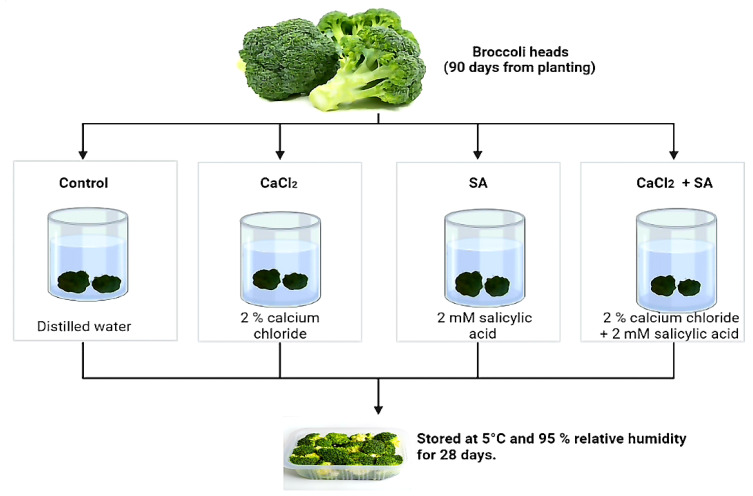
Treatments schema and their concentrations.

Broccoli heads (cv. Imperial) were harvested at the commercial maturity stage (90 days from planting) from a private farm in Giza, Egypt, and transferred within three hours to the laboratory. Healthy heads, free from any defects or insect injuries, with high quality (compact and dark green) were selected for use in this experiment. Broccoli florets were randomly divided into four groups. Every group was immersed in the following solution for 15 min at room temperature (23 °C): 2% calcium chloride, 2 mM salicylic acid (SA), 2% CaCl_2_ + 2 mM SA, and distilled water (control). Florets were allowed to dry at room temperature for one hour and packed in polyethylene trays (clamshells), then stored at 5 °C and 95% relative humidity for 28 days, as shown in Figure 1. The average weight of every tray was 220–230 g, and three replicates were used for each treatment. The following physical and chemical attributes were measured every 7 days.

### 2.2. Weight Loss, Appearance Score, Chlorophyll, and Carotenoids

Florets were weighted immediately after drying and at each sampling time to measure weight loss. The difference between initial weight and sample weight was used to calculate percentage weight loss according to Ali and Abdel-Aziz [27]. The sensory score (appearance) of broccoli florets was conducted by a panel consisting of four trained members (two women and two men) after each sampling time. A sensory score was recorded according to Goa et al. [28]. Appearance and visual quality were determined with a 5–1 scale, where 5 = extremely fresh and marketable (free from any color changes), 3 = fresh and marketable with a slight change in green color (minimum accepted quality), and 1 = moderate color change and unmarketable. To determine the content of chlorophyll and carotenoid in broccoli florets; 1 g of the sample was extracted in 10 mL of *N*,*N*-dimethylformamide for 48 h. Then, the extract was filtered by Whatman filter paper 1 and measured by the spectrophotometer at 470, 647, and 663 nm. The results were expressed as mg/g FW for chlorophyll content and mg/100 g FW for carotenoids according to Moran [29].

### 2.3. Vitamin C, Total Phenolic, and Flavonoids

Vitamin C content was determined in broccoli florets by the titration method according to the method described previously by Shehata et al. [30]. In brief, 10 g of raw material was homogenized for 10 min in 90 mL of oxalic acid (6%). Afterward, 25 mL of filtrated solution was titrated by 2,6–dichlorophenol indophenol, and the results were expressed as mg/100 g FW. Total phenolic was determined by using the Folin–Ciocalteu colorimetric method according to Singleton and Rossi [31]. In brief, ethanol solvent was used to extract total phenolic from fresh broccoli florets (5 g/100 mL) for 20 min. Then, a spectrophotometer (model UV-2401 PC, Shimadzu, Milano, Italy) was used to determine the content of phenolic compounds at 765 nm. The results were expressed as mg of gallic acid equivalent/100 g FW. To determine flavonoids in fresh broccoli florets, the method of El-Beltagi et al. [32] was followed.

### 2.4. Glucosinolates, Sulforaphane, and Peroxidase

Glucosinolates were determined according to the methods of Bjerg et al. [33] and Bjerg and Sorensen [34]. In brief, glucosinolate concentration was calculated by using glucobarbarin as an internal standard. Then, 0.2 g of sample was spiked with a 100 µL standard solution containing 5.0 µmol·mL^−^^1^ of sinigrin and glucobarbarin. To homogenize the previous mix, 5 mL of boiling methanol (70%) was used three times for 2 min using the Ultra-Turrax Homogenizer (Ika-Labortechnik, Staufen, Germany). Then, samples were centrifuged and concentrated to dryness in vacuo. The residue was dissolved in 2 mL of deionized water for HPLC analysis of glucosinolates.

For sulforaphane determination, the methods of Gu et al. [35] and Han and Row [36] were followed. In brief, 0.2 g of sample was subjected to serial dilution (1:20, 1:30, 1:40, 1:50, and 1:60) with acidic water (pH 3.0). The previous samples were incubated at 50, 55, 60, 65, and 70 °C for 1, 2, 3, 4, and 5 h. Immediately after incubation, 40 mL of dichloromethane was added, and the mixture was vortexed and filtered through a 0.45 mL membrane. HPLC (High-Performance Liquid Chromatography) analyses were carried out by injecting a 20 mL aliquot onto a Waters E2695 Liquid Chromatograph (Waters Crop., Milford, MA, USA) connected to a model 2998 (PAD) photodiode array detector.

Peroxidase activity was determined as described previously [4]. Briefly, 10 mL of extraction buffer (50 mM phosphate buffer, pH 7, containing 0.5 mM EDTA and 2% PVPP (*w*/*v*)) was used to grind broccoli samples. Then, the previous samples were centrifuged for 20 min at 21,925 rpm. The method of In et al. [37] was used to determine the peroxidase activity.

### 2.5. Antioxidant Activity (%) Using DPPH

DPPH antioxidant capacity (%) was measured according to Awad, et al. [38], with slight modification. In brief, 0.1 mL of previous ethanol broccoli extract was taken and mixed with 3.9 mL of DPPH solution. The absorbance of the sample and control (0.0024 mg/100 methanol DPPH solution) was measured at 520 nm after incubation in the dark at room temperature for 30 min using a spectrophotometer (model UV-2401 PC, Shimadzu, Milano, Italy). The result was calculated according to the following equation: antioxidant capacity % = [(absorbance of control − absorbance of sample)/absorbance of control × 1000].

### 2.6. Statistical Analysis

Data were subjected to analysis of variance using SPSS software (Ver. 20, SPSS Inc., Chicago, IL, USA). Means of different treatments were compared by the Duncan test at 5% (LSD). The values are presented as means with their standard error. Pearson’s correlation test was performed by SPSS software.

## 3. Results

### 3.1. Weight Loss, Appearance Score, Chlorophyll Content, and Carotenoids

**Figure 2:**
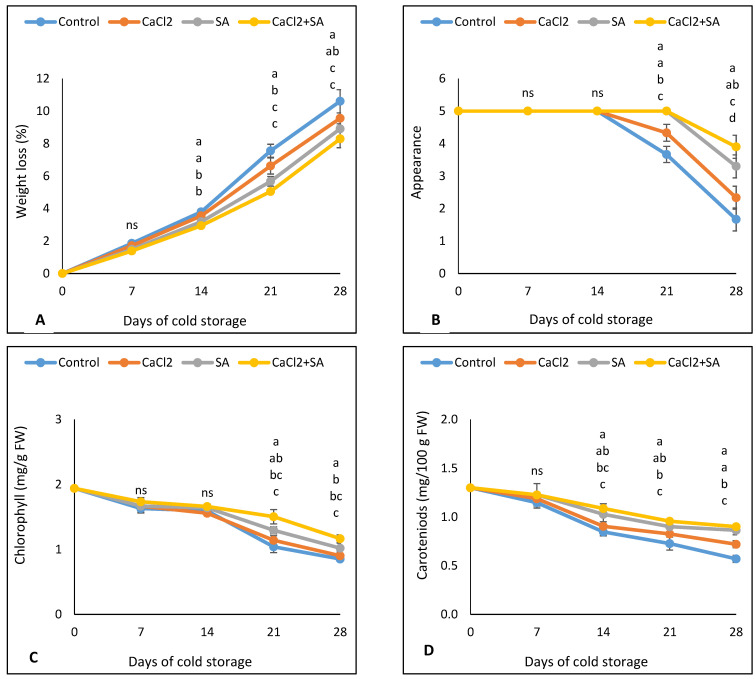
Effect of SA and CaCl_2_ and their combination on (**A**) weight loss, (**B**) appearance, (**C**) chlorophyll content, and (**D**) carotenoids of broccoli florets stored at 5 °C for 28 days. Values are means ± SE from three replicates (*n* = 3). Same letter means no significant differences between the values (*p* < 0.05) according to Duncan test.

Compared to the control, the broccoli florets treated with SA and CaCl_2_ or their combination were effective in reducing weight loss after 14 days until the end of refrigerated storage (Figure 2A).

SA was more effective than CaCl_2_ at reducing weight loss. After 28 days of storage, weight loss in the control was 21.86, 16.02, and 9.89% higher than it was in SA + CaCl_2_, SA, and CaCl_2_ treatments, respectively. A negatively strong correlation was observed between weight loss and all tested parameters (except peroxidase activity) according to Pearson’s coefficient correlation (Table 1).

**Figure 3:**
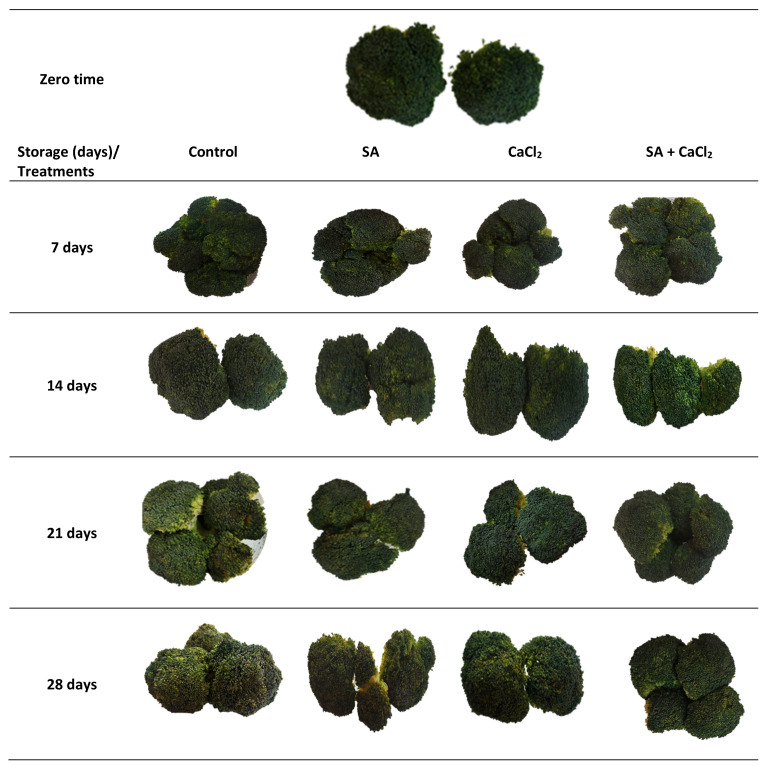
Effect of SA and CaCl_2_ and their combination on the appearance and visual quality of broccoli florets stored at 5 °C for 28 days.

The appearance score rating of broccoli florets displayed a drastic decline after 14 days of storage until the end of the storage period (Figure 2B and Figure 3). However, the quality ranking of SA, CaCl_2_, and their combination treatments remained higher than the control. Control and CaCl_2_ samples showed a limited acceptance quality (less than score 3) after 28 days of refrigerated storage.

A linear decrease in the chlorophyll content of broccoli florets was observed during the refrigerated storage for all treatments (Figure 2C). There was no significant difference in chlorophyll content between treated broccoli florets and non-treated broccoli florets until 14 days of storage at 5 °C. However, after 21 and 28 days of the storage period, SA and SA + CaCl_2_ treatments suppressed the decrease of chlorophyll content compared with the control and CaCl_2_ treatments. After 28 days of storage, chlorophyll content in the control was 27.35, 16.66, and 5.56% lower than it was in SA + CaCl_2_, SA, and CaCl_2_ treatments, respectively. A positive correlation was observed between chlorophyll content and vitamin C, phenolic content, carotenoids, glucosinolates, and flavonoids, while a negative correlation was observed between chlorophyll content and peroxidase activity (Table 1).

As shown in Figure 2D, a linear decrease in the carotenoids of broccoli florets was observed during the cold storage for all treatments. There was no significant difference in carotenoids content between all treatments until 7 days of cold storage. However, at 14 days of storage until the end of the storage time, carotenoids content was significantly higher in the broccoli florets treated with SA, CaCl_2_, and their combination than in the controls. At the end of the storage period, carotenoids in the control were 36.66, 33.72, and 20.83% lower than they were in the SA + CaCl_2_, SA, and CaCl_2_ treatments, respectively.

### 3.2. Total Phenolic Compounds, Vitamin C, Flavonoids, and Glucosinolates

**Figure 4:**
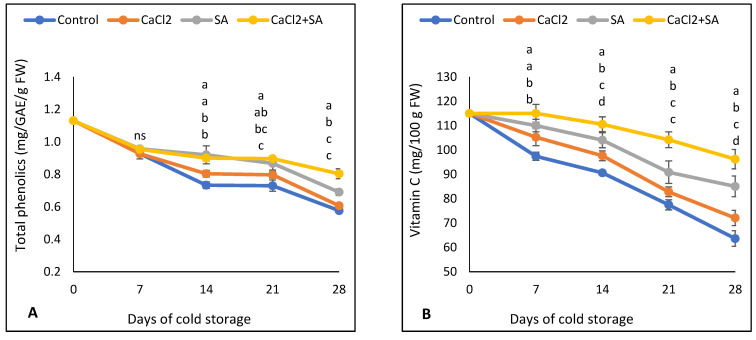

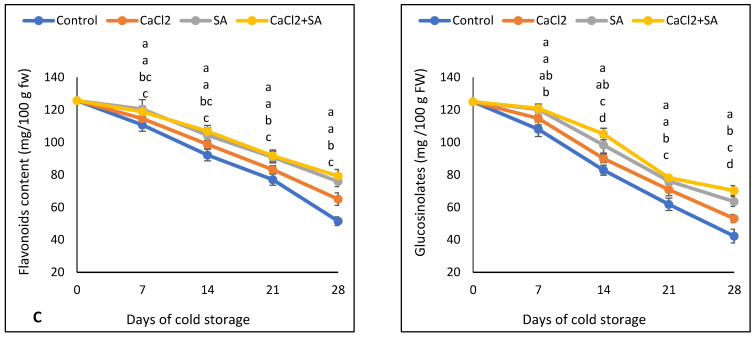
Effect of SA and CaCl_2_ and their combination on (**A**) total phenolic, (**B**) vitamin C, (**C**) flavonoids, and (**D**) glucosinolates of broccoli florets stored at 5 °C for 28 days. Values are means ± SE from three replicates (*n* = 3). Same letter means no significant differences between the values (*p* < 0.05) according to Duncan test.

The content of total phenolic compounds in broccoli florets for all treatments showed a decrease from the beginning until 14 days of storage and then remained constant until 21 days and finally decreased (Figure 4A). The total phenolic content in broccoli florets treated with SA and CaCl_2_ + SA treatments was greater than in the control and CaCl_2_ treatments. At the end of the storage time, the CaCl_2_ + SA and SA treatments reduced the losses in total phenolic compounds by 27.50 and 15.94%, respectively, compared to the control treatment. A positive correlation between phenolic compounds and all parameters was recorded (Table 1).

As presented in Figure 4B, all treatments showed a rapid decrease in vitamin C during the whole period of the cold storage; however, all treatments suppressed the losses of vitamin C compared with untreated broccoli florets. After 28 days of storage, vitamin C in the control was 33.87, 25.17, and 11.71% lower than it was in SA + CaCl_2_, SA, and CaCl_2_ treatments, respectively. Our results showed a positive correlation between vitamin C and all other parameters, except peroxidase activity, which had a slight negative correlation (Table 1).

Flavonoids content decreased rapidly during the cold storage in all samples (Figure 4C). Levels of flavonoids were similar in both SA and CaCl_2_ + SA treatments during all storage periods and were higher than CaCl_2_ and the control, respectively. After 28 days of cold storage, the flavonoid content of the broccoli florets treated with SA + CaCl_2_, SA, or CaCl_2_ was 35.44%, 32.13%, or 20.78% higher than the control treatment. Glucosinolates decreased rapidly with storage time (Figure 4D). However, all treatments showed significantly higher values of glucosinolates after 14 days until the end of storage time compared with the control. Samples treated with SA + CaCl_2_ showed higher glucosinolate content than SA and CaCl_2_.

### 3.3. Sulforaphane, Peroxidase Activity, and Antioxidant Activity

**Figure 5:**
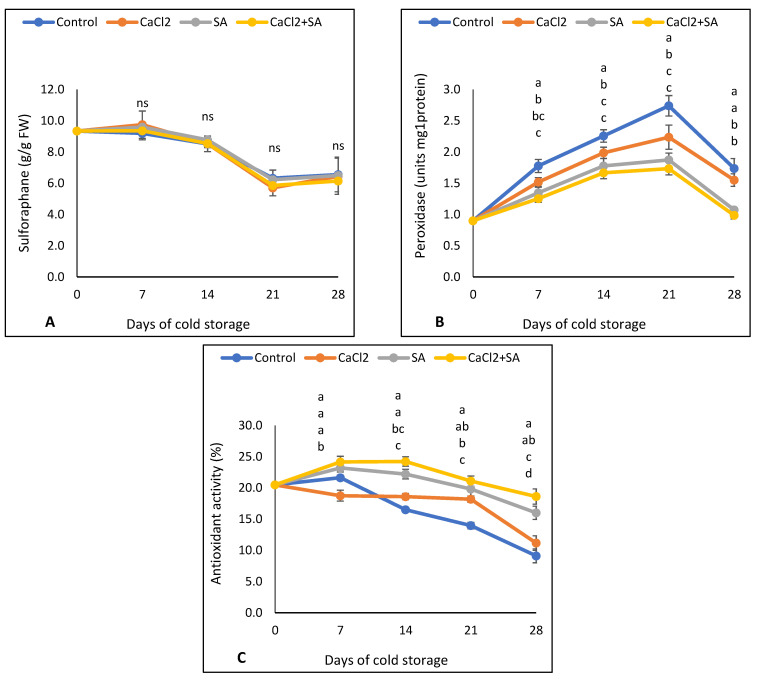
Effect of SA and CaCl_2_ and their combination on (**A**) sulforaphane, (**B**) peroxidase activity, and (**C**) antioxidant activity of broccoli florets stored at 5 °C for 28 days. Values are means ± SE from three replicates (*n* = 3). Same letter means no significant differences between the values (*p* < 0.05) according to Duncan test.

A linear decrease in the sulforaphane content in broccoli florets was recorded in all treatments during storage time. There was no significant difference between all treatments and the control (Figure 5A). The sulforaphane contents in samples were decreased from 9.32 at zero time to 6.54 µg/g FW after 28 days of storage.

Peroxidase activity in all samples showed an increase from the beginning until 21 days of storage and then decreased (Figure 5B). During the storage period, the highest peroxidase activity was observed in the control samples, followed by CaCl_2_ treatment, while the SA + CaCl_2_ and SA treatments showed the lowest peroxidase activities. Peroxidase activity correlated positively with vitamin C and carotenoids, according to Pearson’s coefficient correlation (Table 1). Antioxidant activity was slightly increased after 7 days of storage and then decreased until the end of the storage period in all treatments (Figure 5C). However, after 21 days of storage until the end of the storage, all treatments showed the highest antioxidant activity compared to the control. Antioxidant activity correlated positively with most other bioactive compounds (vitamin C, total phenolic compounds, flavonoids, and carotenoids), while it correlated negatively with weight loss according to Pearson’s coefficient correlation (Table 1).

### 3.4. Correlation Study

**Figure 6:**
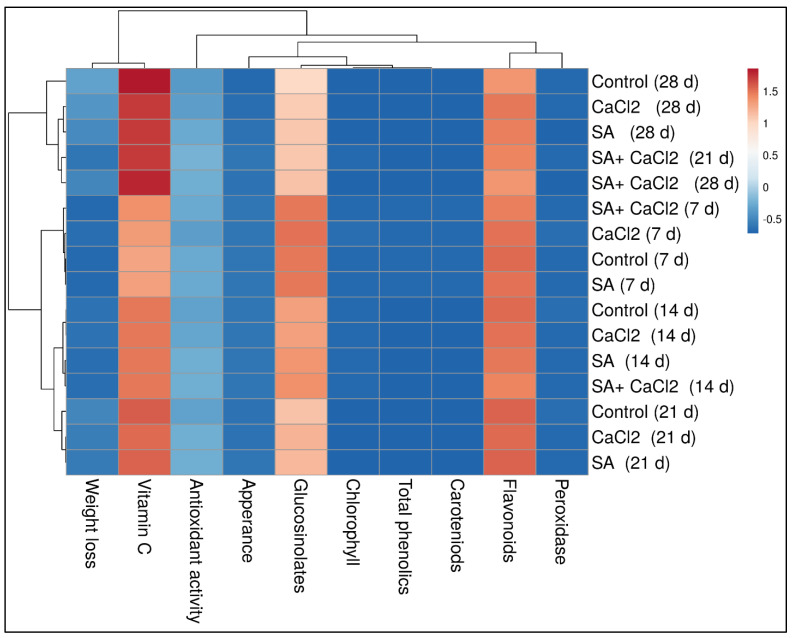
Two-dimensional heatmap visualization shows the interaction between the postharvest exogenous SA and CaCl_2_ treatments and both the measured parameters measured in this study. Lower numerical values are colored blue, whereas higher numerical values are colored red.

Pearson’s correlation study (Table 1) and heatmap (Figure 6) show the changes in bioactive properties of broccoli florets during storage were performed. Pearson’s correlation study and heatmap were presented to further integrate and visualize the findings and data of our study. Significant and insignificant correlations are presented in Table 1. In the heatmap (Figure 6), all the measured parameters of treated broccoli florets during cold storage periods can be seen.

## 4. Discussion

### 4.1. Weight Loss, Appearance Score, Chlorophyll Content, and Carotenoids

The most important factor determining the quality and marketable of horticulture crops is weight loss. Thus, all pre- and postharvest applications that decrease water loss lead to better quality. In this study and in a previous study [4], water loss increased with increasing storage time due to water evaporation, transpiration, and the respiration of broccoli florets [39]. Our results showed that either SA or CaCl_2_ reduced weight loss during storage (Figure 2A). The postharvest application of SA has been used to reduce water loss in horticulture crops such as strawberries [40] and kiwifruits [41]. Reduced weight loss by SA treatment could be due to its inhibitory effects on ethylene biosynthesis [12], which leads to a decrease in respiration rate [42]. Additionally, SA could decrease weight loss by forcing stoma closure [43]. The CaCl_2_ treatment can also reduce weight loss by decreasing the respiration rate [42]. Furthermore, the CaCl_2_ treatment may promote the formation of calcium pectate hydrogel, which is responsible for water retention and cell toughness [44]. Additionally, Kazemi et al. [41] found that the SA + CaCl_2_ dipping treatment decreased weight loss in kiwifruits during cold storage, which supports our hypothesis in this study.

Excellent appearance and deep green color (chlorophyll content) are the most important visual quality parameters of broccoli florets. Thus, yellowing reduces shelf-life and accelerates the senescence of broccoli florets. In this study, the SA application maintained the chlorophyll content, which could be due to its role in decreasing the respiration rate and ethylene biosynthesis [12] and delaying ripening and senescence [45]. Our results are in agreement with Wei et al. [46] and Turkyylmaz et al. [47], who found that SA application maintained the chlorophyll content of asparagus shoots and green beans. The role of CaCl_2_ application for maintaining the appearance and chlorophyll content of broccoli florets could be due to its role in decreasing the respiration rate [48].

Carotenoids are the most abundant pigments in nature, with excellent antioxidant effects and the ability to treat chronic diseases in humans [49]. In this study, SA and CaCl_2_ treatments maintained the level of carotenoids in broccoli florets during cold storage (Figure 2D). Our results agree with Supapvanich and Promyou [15], who found that carotenoids in papaya fruit increased by 2 mM of SA treatment. Robert et al. [50] suggested that CaCl_2_ application reduces the loss of carotenoids during cold storage via respiration reduction.

### 4.2. Total Phenolic Compounds, Vitamin C, Flavonoids, and Glucosinolates

Even under cold storage conditions, significant declines in phytochemicals content have been observed in broccoli florets after harvest [51]. SA is capable of maintaining bioactive components with antioxidant effects in horticultural commodities [52]. For example, the application of 1.0 mmol/L SA significantly reduced the loss of phenolic compounds in asparagus shoots [46]. In addition, SA application activated the biosynthesis of active compounds such as ascorbic acid, phenolic compounds, and flavonoid content [53,54,55,56]. The foliar application of 500 μM of SA maintained the chlorophylls, total carotenoids, and total phenolic contents of coriander [56]. In this study, the CaCl_2_ treatment showed higher phenolic compounds than the control treatment, but the difference was not significant (Figure 4A). However, the SA + CaCl_2_ treatment preserved the phenolic compounds, demonstrating the importance of calcium application in preserving the phenolic compound. Ruiz et al. [57] mentioned the role of calcium in the activation of enzymes such as phenylalanine ammonia-lyase, polyphenol oxidase, and peroxidase, which are responsible for the metabolism of phenolics. In addition, Perucka and Olszówka [58] found that phenolic compounds were increased in lettuce by the application of 0.2 mol/L CaCl_2_.

Vitamin C is a natural antioxidant that could help to reduce the risk of cancer by scavenging reactive oxygen species (ROS) in the human body [59]. However, it is rapidly decreasing in fresh horticultural products due to a variety of circumstances, including refrigerated storage. As a result, it is important to maintain vitamin C content throughout the period of cold storage using an eco-friendly application. Our results showed that both individual SA and CaCl_2_ or their combination were highly effective for preserving vitamin C in broccoli florets during cold storage (Figure 4B). The same result has been recorded in previous work for treated strawberry fruit with the combination of SA and CaCl_2_ [42]. In addition, SA treatment at a rate of 1.0 mmol/L SA was found to be a promising application in preventing vitamin C degradation in asparagus shoots [46]. The reduction in vitamin C loss by SA could be due to its role in activating ascorbate peroxidase activity, which results in a higher accumulation of vitamin C in fresh products [60]. Other research has suggested that high vitamin C contents by SA treatment could be related to the acceleration of biosynthetic dehydroascorbate [53]. The role of calcium in improving the quality of fresh fruits and vegetables has been established earlier [61]. The higher vitamin C level in broccoli florets could be related to the fact that calcium has a promoter effect on vitamin C content [62].

Flavonoids from plant sources, mainly in the skin of fruits, are classified as polyphenolic compounds which have the ability to scavenge free radicals [63]. A decline in flavonoids was observed during cold storage in all treatments (Figure 4C). However, SA and CaCl_2_ or their combination retarded this decline. Our results agree with Dokhanieh et al. [13], who found that treatment with 2 mM of SA significantly increased the accumulation of flavonoids in cornelian cherry fruits during refrigerated storage. SA has an influence on the photosynthetic rate, which could promote the biosynthesis and accumulation of carotenoids [64]. In this study, CaCl_2_ treatment conserved flavonoids during cold storage compared with control samples (Figure 4C). This, in turn, could be due to the effect of calcium application on stimulating the biosynthesis pathways of flavonoids [65].

The anticarcinogenic effects of glucosinolates in broccoli plants have been reported in several works, such as Fahey et al. [65]. In this study, both SA and CaCl_2_ treatments conserved glucosinolates content in broccoli florets during cold storage (Figure 4D). Previous work suggested that CaCl_2_ enhanced glucosinolates biosynthesis, resulting in higher glucosinolates content [66]. Glucosinolates in broccoli florets were enhanced by SA, which could be due to the role of SA in delaying ripening and yellowing, which resulted in a lower loss in glucosinolates content.

### 4.3. Sulforaphane, Peroxidase Activity, and Antioxidant Activity

Our results indicated that sulforaphane was not changed by SA, CaCl_2_, or their combination. In contrast with our results, Zhuang et al. [67] found an increase in sulforaphane content in broccoli sprouts by foliar CaCl_2_ application. The difference with this study could be explained by the difference in the method of CaCl_2_ application or the difference in the content of sulforaphane in florets compared to sprouts. Further studies into the effects of SA and CaCl_2_ on sulforaphane content in broccoli plants are required.

It has been reported that peroxidase activity enhances resistance in plant tissues, such as exposure to unfavorable conditions [68]. In our study, the reduction in peroxidase activity was observed in the SA and CaCl_2_ applications (Figure 5). Our results are in accordance with Lu et al. [69], who reported that peroxidase activity in pineapple fruit was suppressed by SA application under cold storage conditions. The reduction in peroxidase activity in the CaCl_2_ treatment could be due to the role of calcium in reducing the respiration rate [48]. In addition, Guo et al. [70] found that peroxidase activity was decreased by SA + CaCl_2_ + 6-Benzylaminopurine treatment in broccoli heads.

Our results in Figure 5B and previous work [25] indicated that antioxidant activities were increased by Ca treatment. In addition, our results are in agreement with Cui et al. [71], who found that the pre-harvest application of SA maintained a higher antioxidant level in apricot fruits compared with the control. Our results in Table 1 show a significant positive correlation between antioxidant activity and other compounds (chlorophyll content, vitamin C, phenolic compound, carotenoids, flavonoids, and glucosinolates). This, in turn, supported our hypothesis that SA maintained the antioxidant activity of broccoli florets by the accumulation of bioactive compounds.

## 5. Conclusions

**Figure 7:**
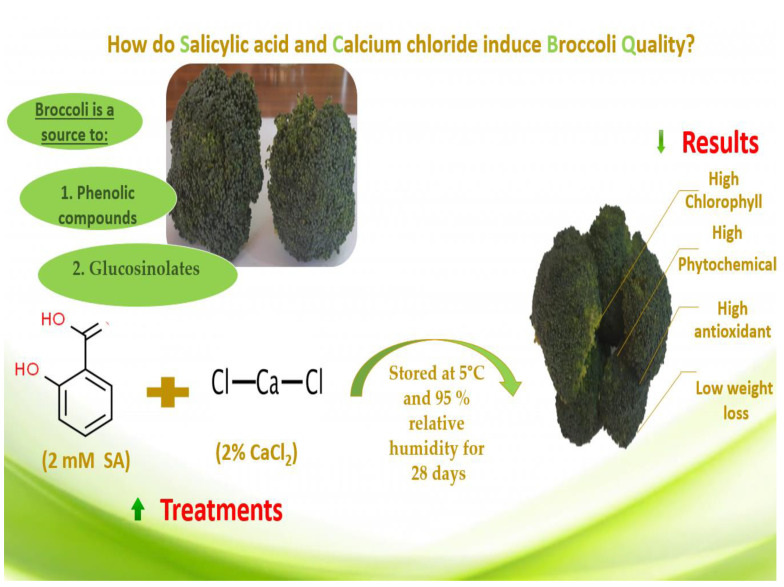
Graphical chart explains the effects of SA and CaCl_2_ on broccoli florets.

The current study showed the effects of SA, Ca, and their combination treatments on broccoli florets’ quality during refrigerated storage at 5 °C for 28 days. The use of SA + Ca combination treatments was more effective than using each treatment separately, while SA outperformed Ca. The postharvest SA + Ca treatment reduced weight loss while preserving the majority of bioactive components such as chlorophyll content, vitamin C, phenolic compound, carotenoids, flavonoids, and glucosinolates, as well as antioxidant activity (Figure 7). This postharvest treatment can be simply applied on a commercial scale to improve quality and increase the shelf life of broccoli florets.

## Figures and Tables

**Table 1:** Pearson’s correlation among the evaluated parameters of broccoli florets.

	Weight Loss	Vit. C	Chlorophyll	Phenolic	Carotenoids	Glucosinolates	Flavonoids	Peroxidase
Vit. C	−0.853 **							
Chlorophyll	−0.954 **	0.887 **						
Phenolic	−0.864 **	0.904 **	0.834 **					
Carotenoids	−0.872 **	0.904 **	0.834 **	0.897 **				
Glucosinolates	−0.960 **	0.894 **	0.916 **	0.885 **	0.948 **			
Flavonoids	−0.972 **	0.907 **	0.925 **	0.915 **	0.934 **	0.985 **		
Peroxidase	0.016	−0.340 *	−0.089	−0.174	−0.383 **	−0.223	−0.155	
Antioxidant	−0.814 **	0.911 **	0.823 **	0.919 **	0.857 **	0.847 **	0.871 **	−0.242

*,**: Correlation is significant at the 0.05 and 0.01 levels, respectively (2-tailed).

## Data Availability

Not applicable.
