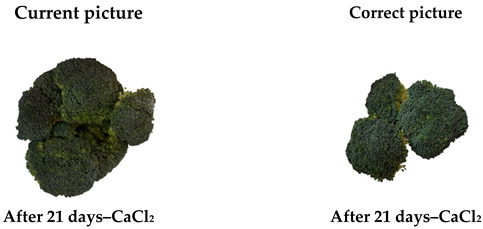# Correction: El-Beltagi et al. Exogenous Postharvest Application of Calcium Chloride and Salicylic Acid to Maintain the Quality of Broccoli Florets. *Plants* 2022, *11*, 1513

**DOI:** 10.3390/plants14192946

**Published:** 2025-09-23

**Authors:** Hossam S. El-Beltagi, Marwa Rashad Ali, Khaled M. A. Ramadan, Raheel Anwar, Tarek A. Shalaby, Adel A. Rezk, Sherif Mohamed El-Ganainy, Samy F. Mahmoud, Mohamed Alkafafy, Mohamed M. El-Mogy

**Affiliations:** 1 Al Bilad Bank Scholarly Chair for Food Security in Saudi Arabia, The Deanship of Scientific Research, The Vice Presidency for Graduate Studies and Scientific Research, King Faisal University, Al-Ahsa 31982, Saudi Arabia; kramadan@kfu.edu.sa (K.M.A.R.); tshalaby@kfu.edu.sa (T.A.S.); arazk@kfu.edu.sa (A.A.R.); salganainy@kfu.edu.sa (S.M.E.-G.); 2 Agricultural Biotechnology Department, College of Agriculture and Food Sciences, King Faisal University, Al-Ahsa 31982, Saudi Arabia; 3 Biochemistry Department, Faculty of Agriculture, Cairo University, Gamma St, Giza 12613, Egypt; 4 Department of Food Science, Faculty of Agriculture, Cairo University, Giza 12613, Egypt; marwa3mrf@agr.cu.edu.eg; 5 Central Laboratories, Department of Chemistry, King Faisal University, Al-Ahsa 31982, Saudi Arabia; 6 Department of Biochemistry, Faculty of Agriculture, Ain Shams University, Cairo 11566, Egypt; 7 Postharvest Research and Training Centre, Institute of Horticultural Sciences, University of Agriculture, Faisalabad 38040, Pakistan; raheelanwar@uaf.edu.pk; 8 Department of Arid Land Agriculture, College of Agricultural and Food Science, King Faisal University, P.O. Box 400, Al-Ahsa 31982, Saudi Arabia; 9 Horticulture Department, Faculty of Agriculture, Kafrelsheikh University, Kafr El-Sheikh 33516, Egypt; 10 Plant Pathology Research Institute, Agricultural Research Centre, Giza 12619, Egypt; 11 Vegetable Diseases Research Department, Plant Pathology Research Institute, Agricultural Research Centre, Giza 12619, Egypt; 12 Department of Biotechnology, College of Science, Taif University, P.O. Box 11099, Taif 21944, Saudi Arabia; s.farouk@tu.edu.sa (S.F.M.); m.kafafy@tu.edu.sa (M.A.); 13 Vegetable Crops Department, Faculty of Agriculture, Cairo University, Giza 12613, Egypt

In the original publication [1], there was a mistake in Figure 3 as published. There is a duplicate of one of the broccoli photos (7 Day and SA is the same of the 21 Day and CaCl_2_). The corrected figure appears below. The authors state that the scientific conclusions are unaffected. This correction was approved by the Academic Editor. The original publication has also been updated.